# A minimal soft tissue damage approach of spondylolysis repair in athletes: preliminary report

**DOI:** 10.1007/s00590-017-1974-0

**Published:** 2017-05-11

**Authors:** Łukasz Bartochowski, Wojciech Jurasz, Jacek Kruczyński

**Affiliations:** 1 Department of General Orthopaedics, Orthopaedic Oncology and Traumatology, University of Medical Sciences, Poznań, Poland; 2 Wiktor Dega Hospital in Poznań, 28 czerwca 1956r. Street Number 135/147, 61-493 Poznań, Poland

**Keywords:** Spondylolysis, Direct pars repair, Surgical technique modification

## Abstract

**Purpose and hypothesis:**

Both spondylolysis and spondylolisthesis come in second place in the causes of pain among athletes. Treatment options include both conservative management and different operative methods. Athletes and adolescents are groups where the priority is to protect tissues from perioperative damage.

**Objective:**

We present our modification of the Buck’s, direct pars repair method, which we believe offers maximum protection of tissues. We used the modified surgical method in young, competitive athletes, in whom non-surgical treatment was not effective.

**Method:**

Eight pars defects in five patients were treated using suggested method. All of them were young males (aged between 13 and 18 years), who practice soccer professionally. We use modified method of direct repair pars through the cannulated screw fixation, first proposed by Buck. Preoperative preparation consists of proper analysis of computer tomography images in multiplanar reconstruction mode: measuring screw length, measurement of inclination angle of the optimal screw trajectory in the frontal and sagittal plane. During the operation, the wire proper direction is performed by usage of the predetermined angles. Starting point for guide wire was also changed to the lower end of the facet. The fusion takes place with a screw of 3 mm diameter. After the operation patient need to use thoracolumbar spinal orthosis as a primary immobilization for 6 weeks and appropriate rehabilitation for another 6 weeks. We used these methods in eight pars fixations.

**Results:**

All of the patients were painless in first week after surgery. All of them underwent total rehabilitation programme and returned to sport.

**Conclusions:**

Direct pars repair using Buck’s method with proposed modification, including adequate radiographic preparation, the use of a thin cannulated screw and changing the point of screw entry, allows precise and safe screw placement, regardless of the size of the bone at the defect site.

## Introduction

Both spondylolysis and spondylolisthesis comes in second place in the causes of pain among athletes [14]. This problem is particularly marked in sports where there are repetitive movements of rotation and hyperextension of the lumbar spine [13]. Treatment options include both conservative management (reduction of activity, splinting, rehabilitation) and different operative methods of healing with damaged pars interarticularis [4, 13]. In 1970, Buck developed a method of direct repair of damaged pars with the use of screws placed on the lower edge of the lamina through the defect and moved upward, in the direction of the upper facet joint. This original method was carried out by open tissue preparation [1]. Its efficacy, in the treatment of spondylolysis, is more than 90% in both athletes and in people not practicing sport. Spondylolysis occurs more frequently in two groups: adolescents and athletes [3]. These are groups where the priority is to protect tissues from perioperative damage. We therefore present our modification of the Buck’s method, which we believe offers maximum protection of tissues by the use of minimally invasive techniques and the use of implants optimally matched to the diameter of the pars interarticularis.

## Methods

### Aims

To develop a method of:

1. Determining the optimum path for screw insertion in preoperative planning in order to minimize the chances of improper entry of the implant.
2. Maximizing the protection of soft tissues (muscles, fascia).
3. Providing a safe margin of bone around the screw.
4. Enabling an early return to full physical activity.
5. Enabling proper screw placement without usage of computer tomography during surgery to avoid overdose of radiation in young athletes.

### Preoperative planning

The indication for surgery was local pain in the lumbar region, coinciding with the place of occurrence of spondylolysis, which persists at least 3 months despite typical conservative treatment. The pain was present when typical moves were performed (hyperextension, rotation), only during a very intense physical activity. Activities of daily living did not trigger any discomfort. Each patient underwent magnetic resonance imaging as part of the diagnostic process, which has not revealed any changes that may be responsible for reported problems. We obtained written informed consent from parents of the minors in each case.

**Fig. 1:**
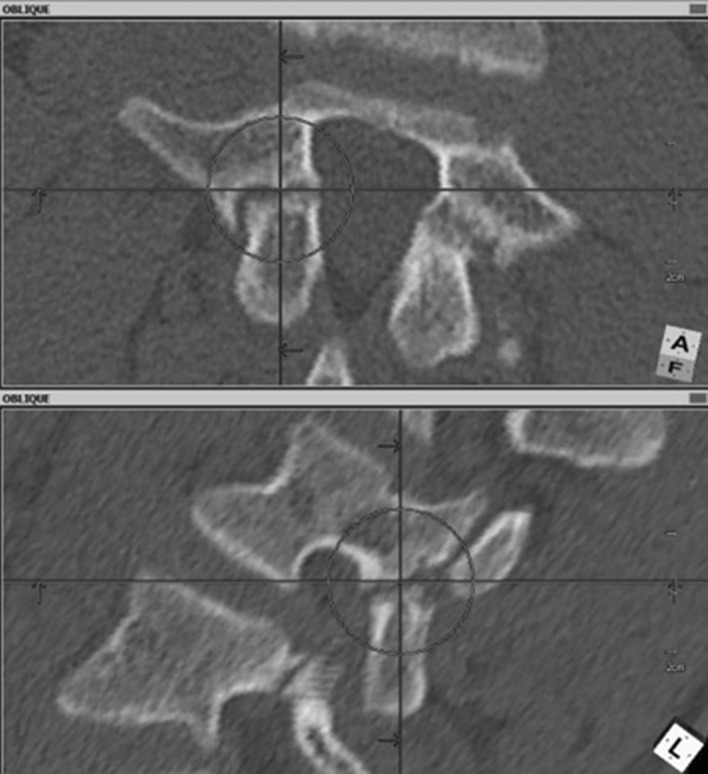
Pars in the multiplanar reconstruction mode set in the view of maximum width

**Fig. 2:**
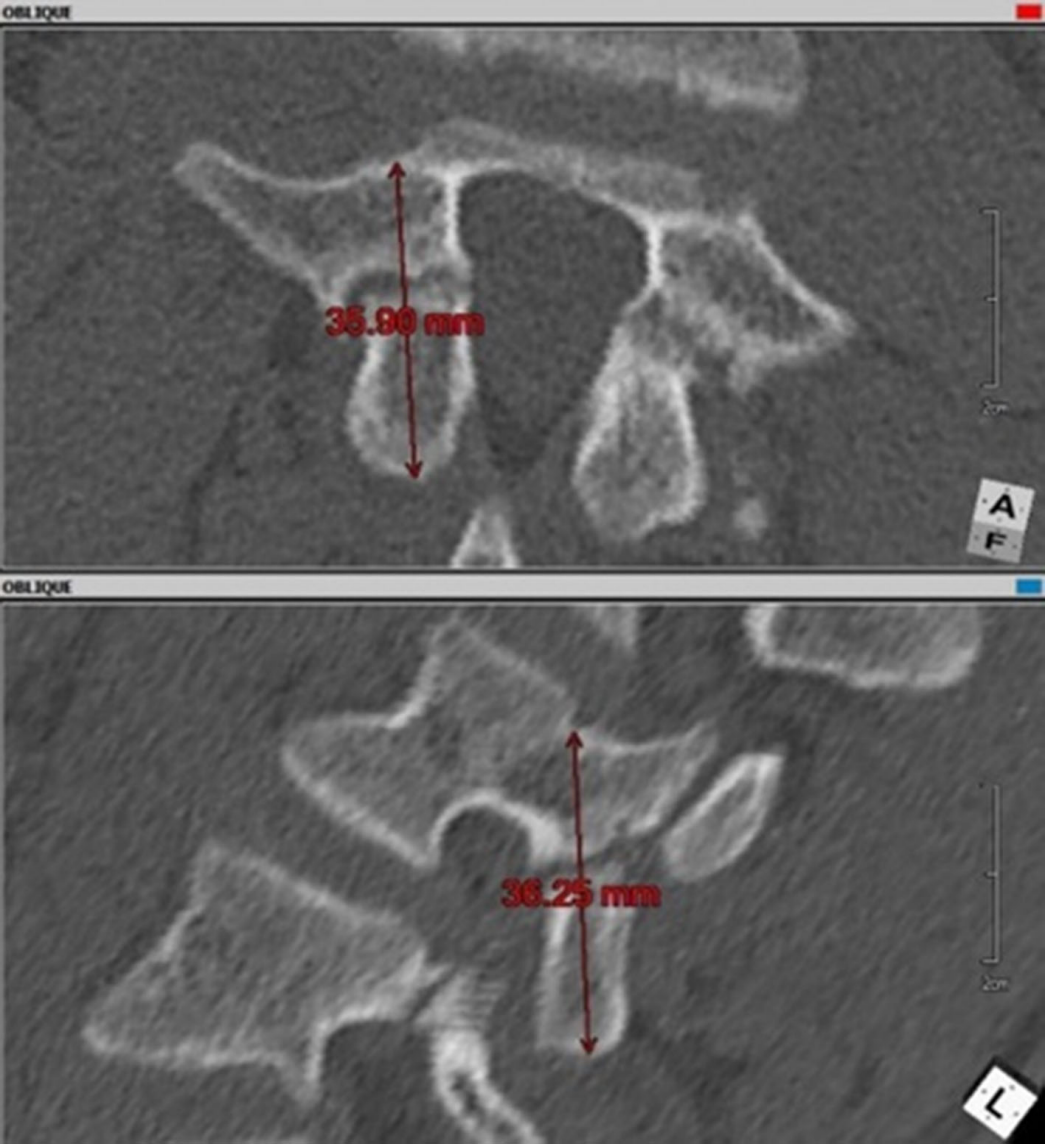
Measurement of maximal length of the screw

**Fig. 3:**
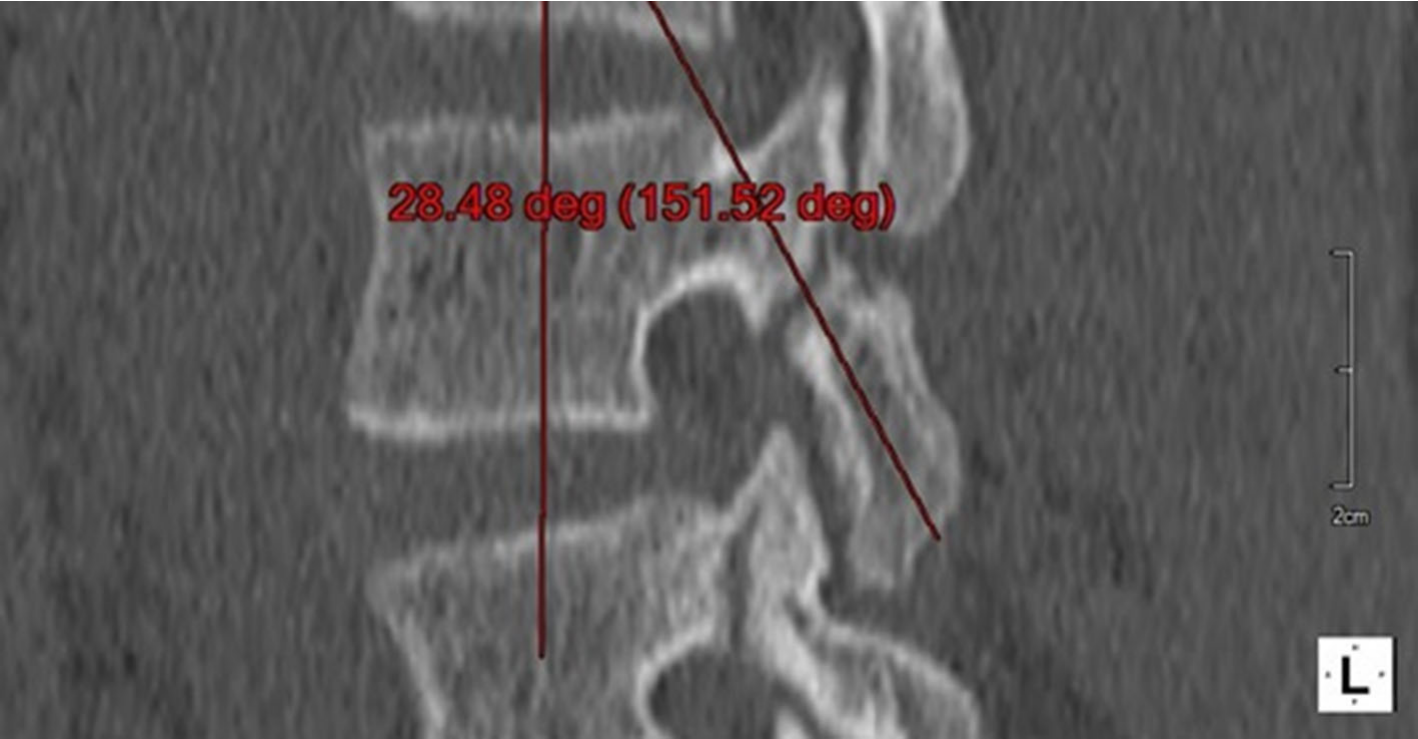
Measurement of angle of the pars inclination in sagittal plane

**Fig. 4:**
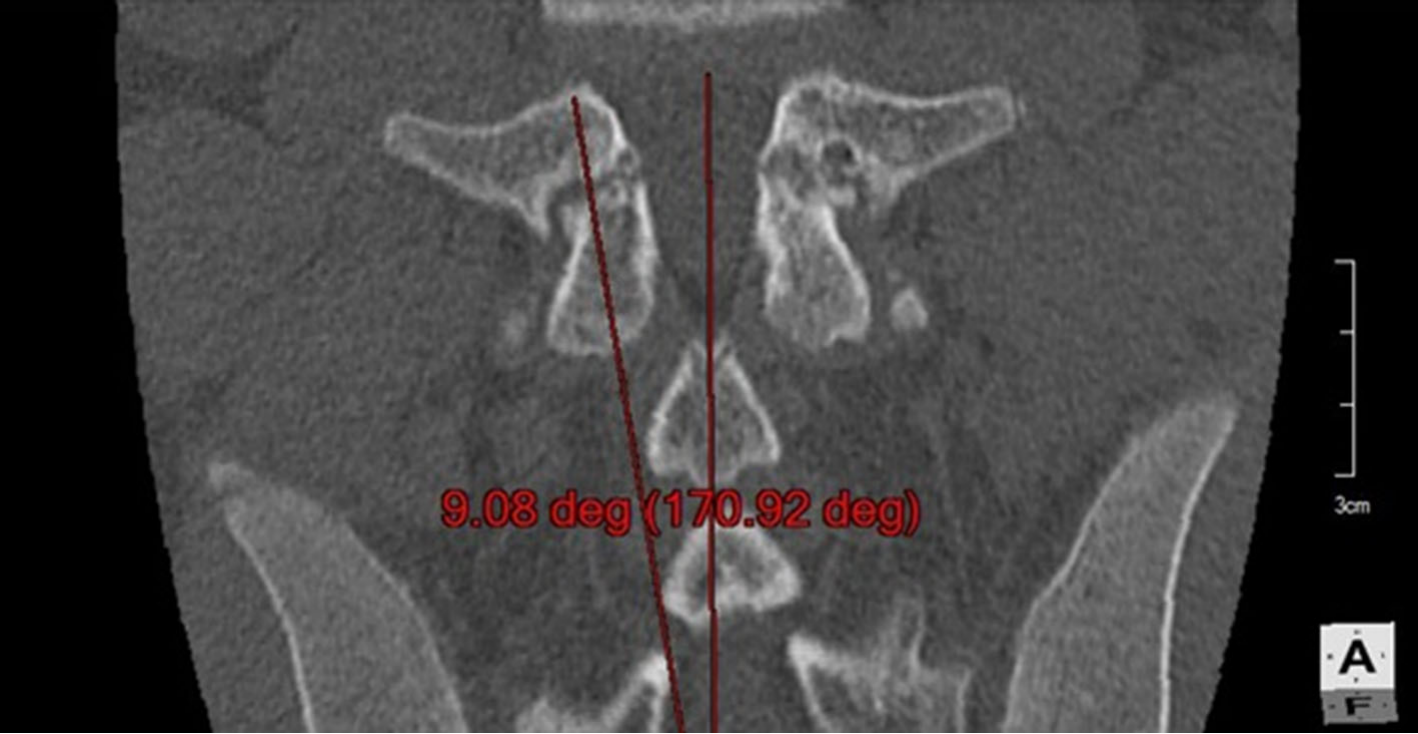
Measurement of angle of the pars inclination in coronal plane

Before surgery, standard computed tomography is performed. Then, in the multiplanar reconstruction modes, planes of maximum width of the pars interarticularis are determined (Fig. 1). This allows the long axis of the pars, which is the optimal path for screw insertion, and the length of screw required, to be measured. The axis screw length is measured (Fig. 2), and inclination angles can be determined in both the sagittal (Fig. 3) and tilted frontal planes (Fig. 4).

### Preoperative preparation

**Fig. 5:**
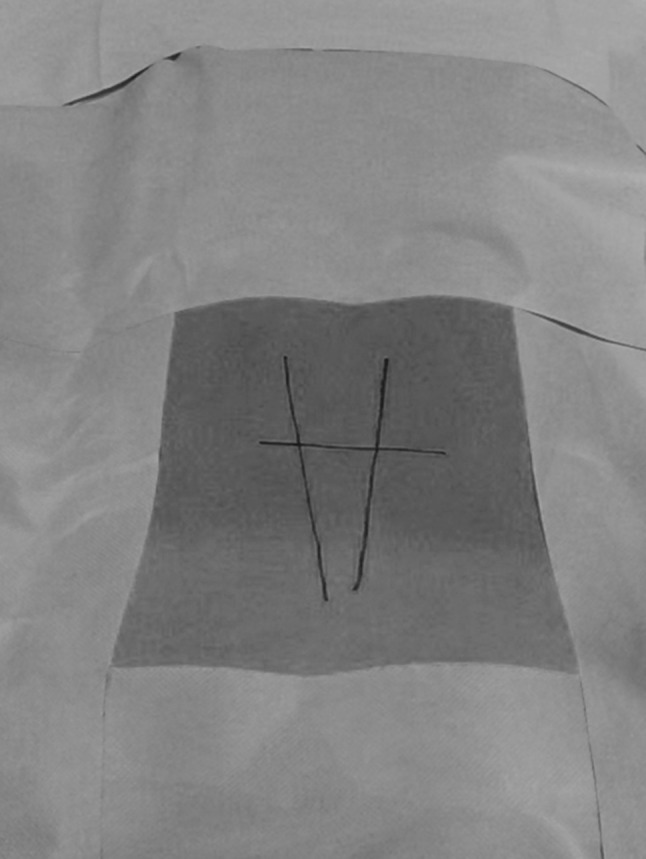
*Vertical lines* show anterio-posterior (AP) view over the pars interarticularis according to predetermined angles. *Horizontal line* shows level of pars defects

**Fig. 6:**
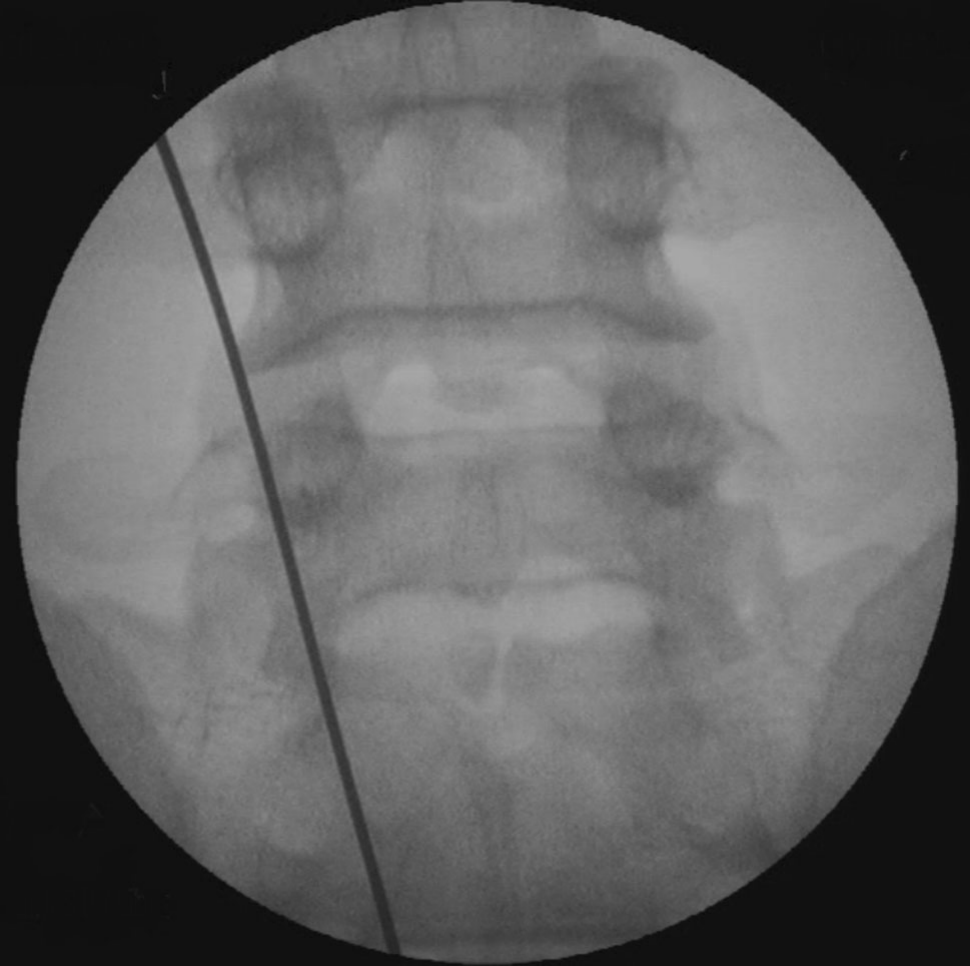
Wire placed over the pars interarticularis in AP view X-ray

During the operation, the patient is positioned prone on a translucent operating table and a C-arm X-ray is used throughout the whole procedure. A marker wire is placed on the patient’s back and an anterio-posterior (AP) view is taken over the pars interarticularis, at the predetermined angle, and a line is drawn on the skin along the wire (Figs. 5, 6).

**Fig. 7:**
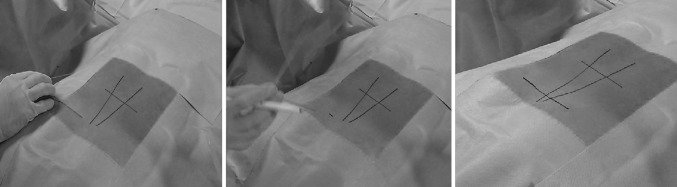
Placing bent wire to determine the skin entry point

**Fig. 8:**
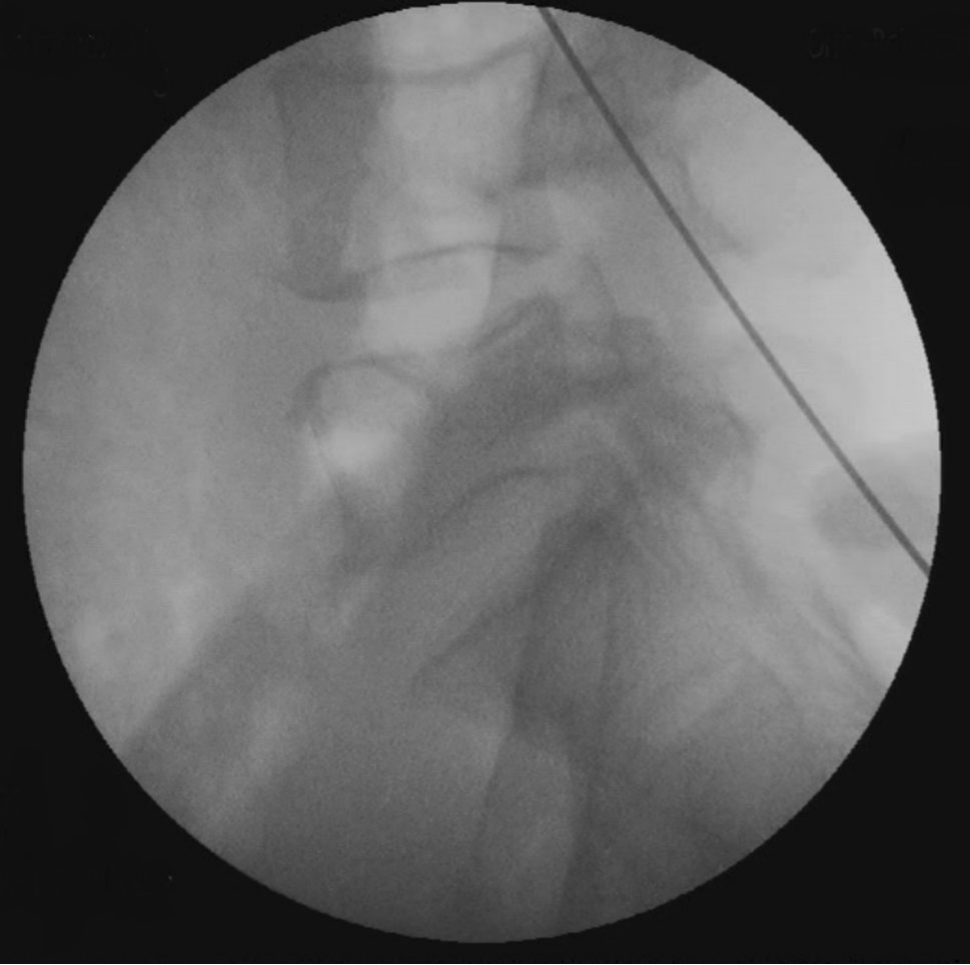
Wire placed over the pars interarticularis on lateral view X-ray

Another wire is bent at a right angle and placed on the patient’s back so that one arm is perpendicular to the long axis of the body and the other is in line with the pars as seen on the lateral view. A line is then drawn along this wire with a skin pencil. The intersection of the lines is the skin entry point (Figs. 7, 8).

Skin and fascia incisions are made. Since 1.1-mm wire is very flexible and is virtually impossible to keep it straight at the desired angle, a cannulated drill with the wire serving as stylet is used. When the tip of the drill crosses the defect, the wire stylet is advanced further. As the tip of the wire reaches the upper facet, the drill is removed and a cannulated screw is inserted over the wire. Drilling, wire insertion and screw advancement are performed under a radiologic oblique view.

### Operation

The patient is positioned prone on the translucent operating table. The pars defect is localized with the C-arm, in AP, lateral and oblique from 30 to 40 degrees planes, adapted to each operated pars, calculated on the basis of the computer tomography image.

A skin incision approximately 1–1.5 cm long is made. The cut in the fascia should be slightly longer cephadally relative to the skin incision. This allows manoeuvring of the guide wire and drill. The guide wire, 1.1 mm diameter, is inserted through the skin incision. Unlike the original Buck method, where the screw was placed from the lower edge of the lamina, in our method the starting point is located at the bottom of the lower facet. Therefore, instead of the original method, it is moved slightly laterally. After the initial entry of the guide wire, it is positioned at the correct angle indicated by the computer tomography. Due to the high flexibility of the guide wire, transmission and maintenance of the proper trajectory can be improved with the use of a drill. After passing the drill and wire through the defect, the drill is withdrawn and the wire is left in place to drive the screw. Fusion is performed using a titanium, partially threaded, self-tapping, 3-mm-diameter screw. The length of the screw is predetermined from the computer tomography.

### Postoperative care

**Fig. 9:**
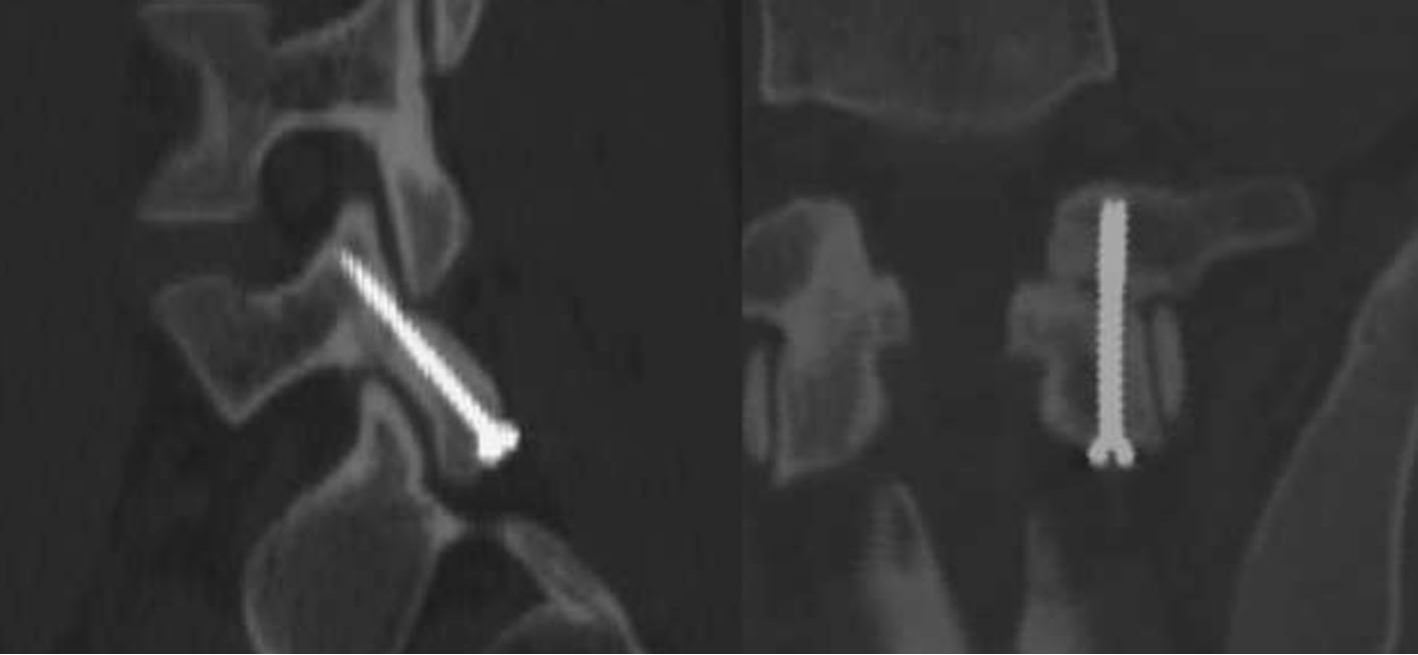
Postoperative CT scan in sagittal (*left figure*) and coronal (*right figure*) plane

All patients underwent postoperative radiological follow-up. Location of screws in both the frontal and sagittal plane was correct: screw ran through the previously predetermined trajectory without exceeding the bone border. Due to the age of patients control CT examination was done in two patients (Fig. 9).

For 6 weeks after surgery, the patient uses a thoracolumbar spinal orthosis (TLSO). Flexion, hyperextension and rotation should be avoided. After the TLSO is discarded, the patient should perform isometric exercises of the abdominal muscles for 2 weeks. Over the next 2 weeks active exercises of the back and isometric exercises of the abdomen (without moving the lumbar spine) shall be performed and over the following 2 weeks active exercises of the back and aerobic exercise (e.g. cycling) should be done. So after 12 weeks the patient can return to normal physical activities, appropriate for the sport concerned. As in other articles about spondylolysis [6, 12], return to the professional sport was possible only when the typical physical activity for a particular sport (for these patients that was soccer) was completely painless.

## Results

We obtained approval for this study from an ethics committee of Poznan University of Medical Sciences. To date, eight pars defects in five patients were treated with the proposed method. All were males who professionally practice soccer. The age range was 13–18 years. In three cases, spondylolysis occurred unilaterally, on one level. In one case, spondylolysis had occurred on both sides on two levels. In one case, spondylolysis occurred on both sides on one level. Optimum screw location was achieved in all cases, i.e. passing from the base of the lower facet to the top of the upper facet without piercing the cortical bone. Pain levels in the first postoperative day were 2–3 on the visual analogue scale (VAS). All the patients were discharged on the first postoperative day. During the first postoperative week, the level of pain in all patients was 0 on VAS. There were no complications. All patients, after a 6 weeks period of immobilization in TLSO and 6-week rehabilitation, returned to sport at the previous level, while remaining completely free from pain and without functional deficits. Because the pain occurred only during intense physical activity, as it was mentioned at the beginning, ODI questionnaire was not applied, due to the lack of evaluation of this type of situation.

## Discussion

Spondylolysis occurs in 6% of the population. The occurrence amongst athletes is much greater, averaging 15% [2, 5, 10]. Non-surgical treatment is effective in almost 90% of people practicing sport professionally or competitively [6]. In others, especially professional athletes, pain associated with spondylolysis can significantly interfere with the achievement of appropriate results. There are currently several methods of obtaining fusion in spondylolysis, without fusing the entire motion segment [3, 7–9, 11]. In comparison with these methods, especially in the population of athletes, Buck’s method is characterized by the highest efficiency [4]. The greatest disadvantage of this method is the need for muscle dissection from the lamina to the facet joint [1]. This causes considerable tissue damage which, combined with a scar generated in the subcutaneous tissues, may limit the optimum functionality of the back muscles. This is particularly undesirable in professional athletes, where even the slightest change in the functionality of tissues may affect the results achieved in sport. For this reason, methods of treatment, which achieve the effects obtained by the Buck method, but which spare tissue through the use of minimally invasive access, have been developed. These use the cannulated screw insertion path as recommended by Buck, after inserting the guide wire [15, 16]. The use of screws with a diameter of 4 or 4.5 mm is described in this methods [15, 16], but according to our measurements, obtained from the preoperative computer tomography, the diameter of the pars interarticularis at the thinnest location ranges from 3.5 mm to 5 mm. We therefore decided to modify the Buck’s method in order to cause minimal tissue damage and to enable the insertion of a 3-mm screw which, while allowing for a safe fixation of the defect, leaves a margin of bone. The main problem encountered in the use of a 1.1-mm guiding wire is its flexibility, which makes it impossible to move the wire seamlessly in one direction while running in bone tissue. The entry point in the bone is located far from the skin incision so that it is difficult to adjust the wire direction once it is in the soft tissues, which is why it is so important to be properly prepared for surgery by analysing CT to define the appropriate angle. During intraoperative fluoroscopy, we can choose the same bone landmarks as in CT and can accurately determine both the skin incision site, to allow a correct start, and the trajectory of the guide wire and drill. Preoperative planning also allows the surgeon to observe the possible obstacles (spinous processes, sacrum), he may encounter when inserting directional wire and screws. In the original Buck’s method, the point of entry of the screw is located on the lower edge of the lamina [4]. Anatomically this place, where yellow ligament adheres, is very narrow. In the open type of surgery, location of this point poses no difficulties, but in the closed method there is a substantial risk of violating the spinal canal. We therefore propose modification of the entry point of the screw, placing it on the lower end of the lower facet. An important aspect of our technique is that the use of fluoroscopy constitutes an improvement over the starting point proposed by Buck. Our results, both radiological and clinical, support the efficacy of the Buck’s method, but our proposed modification reduces the amount of soft tissue damage.

## Conclusions

For patients who do not respond to conservative treatment, direct pars repair using Buck’s method with our modification, including adequate radiographic preparation, the use of a thin cannulated screw and changing the point of screw entry, allows precise and safe screw placement, regardless of the size of the bone at the defect site. Good functional results and a lack of complications support the clinical efficacy of our modifications.
